# A Framework for Agricultural Pest and Disease Monitoring Based on Internet-of-Things and Unmanned Aerial Vehicles

**DOI:** 10.3390/s20051487

**Published:** 2020-03-08

**Authors:** Demin Gao, Quan Sun, Bin Hu, Shuo Zhang

**Affiliations:** 1 College of Information Science & Technology, Nanjing Forestry University, Nanjing 210037, China; sunquan@njfu.edu.cn (Q.S.); zhangshuo@njfu.edu.cn (S.Z.); 2 Department of Computer Science and Engineering, University of Minnesota, Minneapolis, MN 55414, USA; 3 College on Information Science & Technology, Nanjing Agriculture University, Nanjing 210095, China; hubin@163.com

**Keywords:** agricultural pests and diseases, internet of things, unmanned aerial vehicle

## Abstract

With the development of information technology, Internet-of-Things (IoT) and low-altitude remote-sensing technology represented by Unmanned Aerial Vehicles (UAVs) are widely used in environmental monitoring fields. In agricultural modernization, IoT and UAV can monitor the incidence of crop diseases and pests from the ground micro and air macro perspectives, respectively. IoT technology can collect real-time weather parameters of the crop growth by means of numerous inexpensive sensor nodes. While depending on spectral camera technology, UAVs can capture the images of farmland, and these images can be utilize for analyzing the occurrence of pests and diseases of crops. In this work, we attempt to design an agriculture framework for providing profound insights into the specific relationship between the occurrence of pests/diseases and weather parameters. Firstly, considering that most farms are usually located in remote areas and far away from infrastructure, making it hard to deploy agricultural IoT devices due to limited energy supplement, a sun tracker device is designed to adjust the angle automatically between the solar panel and the sunlight for improving the energy-harvesting rate. Secondly, for resolving the problem of short flight time of UAV, a flight mode is introduced to ensure the maximum utilization of wind force and prolong the fight time. Thirdly, the images captured by UAV are transmitted to the cloud data center for analyzing the degree of damage of pests and diseases based on spectrum analysis technology. Finally, the agriculture framework is deployed in the Yangtze River Zone of China and the results demonstrate that wheat is susceptible to disease when the temperature is between 14 °C and 16 °C, and high rainfall decreases the spread of wheat powdery mildew.

## 1. Introduction

Due to population growth and social development, world food demand is expected to double by 2050 [1], but it is currently challenging to increase food production because of falling water levels, climate change, arable land reduction, and pests and diseases [2]. Pests and diseases have always been among the critical factors that restrict the increase of grain production [3], causing substantial economic losses to agriculture. According to the statistics of the Food and Agriculture Organization of the United Nations (FAO), global grain production will be reduced by 10–16% annually owing to the occurrence of crop pests and diseases. In China, investigation shows that pests and diseases cause about 40 million tons of grain loss each year [4].

For detecting the occurrence of pests and diseases on farms, remote-sensing technologies (e.g., satellites and drones (in this paper, we will use “drone” and “UAV” interchangeably)), are employed to find insect pests and inform farmers of the state-of-affairs promptly [5]. Agricultural detection technology depending on satellites, called high-altitude remote-sensing technology, has the advantages of the extensive monitoring area, fine timeliness, short revisit period, and low cost [6]. On the one hand, a satellite device can cover a large area and is suitable for a wide range of disaster monitoring. On the other hand, satellite technology is susceptible to weather and has a low spatial resolution, making it challenging to meet the need for pest and disease monitoring in agricultural fields [7]. Now, the remote-sensing technology with low-altitude (e.g., drones) has the characteristics of high flexibility and image definition [8], which can meet the requirements of pest and disease monitoring for crops.

Currently, the detection technology depending on drones or UAV devices, named low-altitude remote-sensing technology, has been applied widely in modern farms [9], which can guarantee a high timeliness of acquired data [10]. When drones are used for detecting the occurrence of pests and diseases, the information about pests and diseases of crops should be standardized and digitized [11]. However, for a drone, due to limited carrying weight and battery capacity in a remote large-scale farm, it faces the problems containing short flight time and frequent battery replacement [12]. Nowadays, these characteristics affect the promotion and application of drones in the modern agricultural field. In this work, for reducing the energy consumption of a drone, its flight route on the farm is planned previously to ensure that the entire farm is checked with the shortest flight distance. At the same time, the drone can utilize wind force to prolong its flight time when it flies on the farm and selects the path with the largest proportion of the downwind distance. Besides, the drone can adjust its flying angle dynamically for saving energy according to different wind directions.

The eruption of pests and diseases is closely related to climate change during the crop growth period [13]. IoT technology is utilized to collect information on entities of interest [14], which provides a convenient method to monitor the growing processes of crops in real time. However, most farms are often far away from infrastructure and face the problem of limited energy supply, which restricts the development and popularization of agricultural IoT technology [15]. At present, the energy harvesting type of IoT technology represented by solar power is gradually applied to the agricultural field [16]. Nevertheless, in the currently used photovoltaic power generation system, fixed solar panels are mainly used for convenience [17]. The solar energy conversion rate is related to the angle between the solar panel and sunlight [18], and it is on the influence of the light angle. In the work, an automatic rotary-device based on angle perception of sun illumination is designed for ensuring that the solar panel is always perpendicular to the sunlight, which ensures that more solar power is converted into electric energy.

The contributions are summarized as follows:

- An automatic rotary-device based on angle perception of sun illumination is designed for ensuring the solar panel is always perpendicular to sunlight and improving the energy-harvesting rate from solar power.
- An IoT framework containing multiple wireless technologies (e.g., LoRa, ZigBee, TVWS) is proposed for collecting information and transmitting the collected data to the base station/gateway.
- A strategy to prolong the flight time of a drone is introduced by planning the flying path with the largest proportion of downwind and ensuring the maximum utilization of wind force.

The remainder of this paper is organized as follows. Section 2 gives a review on the existing strategies for monitoring agricultural pests and diseases. Section 3 presents our method and design in detail. Section 4 contains experimental results and Conclusion is in Section 5.

## 2. Related Work

Pests and diseases of crops have always been an important factor hindering agricultural development [19]. A great quantity of works have been proposed for monitoring agricultural pests and diseases, i.e., early warning system of diseases and pests based on wireless sensor network [20,21] and remote video interactive systems [22]. In [23], a pest and disease reporting system is established to investigate and report on various crops such as potatoes, soybeans, rice, and sweet potatoes. In [24], the authors review the influence of pests and diseases to global tomato production. Denmark [25] has developed a pest and disease monitoring system to monitor seven crops such as winter wheat and spring barley, and developed a plant protection information system to integrate pesticides, pests, plant protection programs and field records.

At present, the mainstream pest and disease monitoring technologies applied internationally can be divided into two categories: IoT technology and remote-sensing technology. The IoT technology has emerged as a significant promising technology [26,27], containing numerous inexpensive sensor nodes randomly scattered over the area of interest to collect information on entities of interest [27,28,29], have been used for wide-ranging applications in the fields of climate simulation monitoring [30], real-time video monitoring of pests and diseases [31], and real-time early warning systems for predicting the occurrence of pests and diseases [32]. For agroclimatic diseases (such as wheat powdery mildew), the establishment of climate models through data collected by small climatic instruments arranged in the field can markedly reduce the incidence of pests of crops [33]. In [34], the authors propose a hardware design architecture of the Wireless Sensor and Actuators Networks (WSANs) for real-world agricultural applications.

Recent advances in remote-sensed imagery and geospatial image processing using unmanned aerial vehicles have enabled the rapid and ongoing development of monitoring tools for crop management and the detecting/surveillance of insect pests and diseases [35]. The real-time video monitoring system for pests and diseases is established publicly by the aid of high-definition cameras [36] on UAVs or mounting racks, which means farmers have to enter the farmland for checking the crops frequently and improve the overall working efficiency [37]. For mapping the overflown environment of farms in point clouds, a Light Detection and Ranging (LiDAR) sensor mounted on an Unmanned Aerial Vehicle (UAV) is introduced in [38], Other examples can be found in [39,40,41] and references therein.

Some monitoring and early warning devices for climatic epidemics of crops have been employed in China, such as the potato late blight real-time monitoring [42] and early warning system adopted by Chongqing (City of China) which relies on sensor devices deployed in the farm to collect important environmental parameters, such as humidity, temperature, wind speed and rainfall [43]. Then, these parameters are uploaded to a cloud data center through wireless networks, and the server uses the pre-established model based on big data analytic methods to analyze the probability of occurrence of plant diseases, and provides an early warning to formers [44]. In [45], the authors perform a review on current studies and research works in agriculture which employ the recent practice of big data analysis, in order to solve various relevant problems.

However, with regard to the current research and application of IoT technology in the agricultural pests-monitoring field, the IoT monitoring technology is not mature enough, and there is still a gap between the standards and demands for large-scale promotion. The factors restricting the application of agricultural IoT technology have not been fully solved yet, e.g., energy supply, data fusion and communication, etc. Moreover, the IoT technology used to monitor pests and diseases has not yet formed a unified standard in terms of interfaces, services and equipment development. At present, most of the existing IoT monitoring technologies are still at the stages of demonstration and experiment, failing to build a complete IoT monitoring platform for pests and diseases.

## 3. System Model

### 3.1. The Agricultural IoT Platform

The occurrence of plant diseases and pests is closely related to many weather paraments [46], such as the pests of crop surviving in some unusual weather conditions. At the same time, rainfall and high humidity will affect the propagation and spread of pathogens significantly [47]. Wind can not only affect the spread of spores, but also increase the risk of pests and diseases for crops. Therefore, for analyzing the reasons causing the occurrence of diseases and pests of crops, a modern agricultural IoT platform is designed for monitoring plant pests and diseases. In the system, the primary role of the agriculture IoT platform is to collect the information and monitor the weather parameters of the farm.

**Figure 1:**
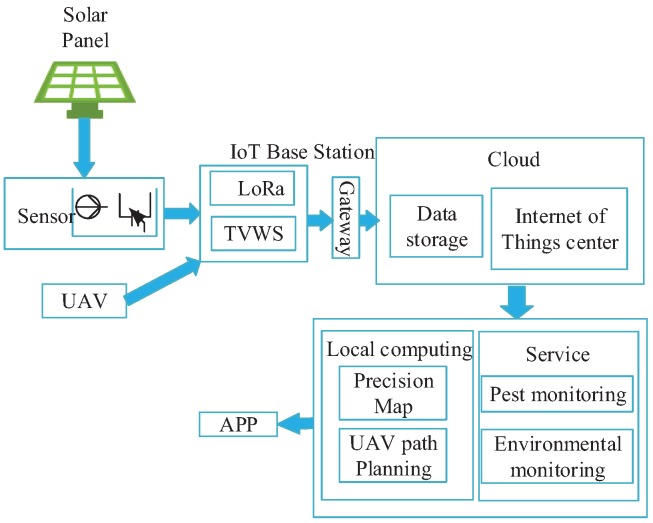
Overview of the agricultural IoT platform.

Considering a farm is open and in outdoor generally, the agricultural IoT platform mainly contains the following parts: energy supplying devices, IoT base stations, gateways, a cloud data center, and APP (Application) software. An overview of the system is given in Figure 1. A smart solar power system based on angle perception of sun illumination is designed to provide power for the platform, the detail of energy supplying system is provided in Section 3.2. The IoT base station is mainly composed of TV White Spaces (TVWS) and LoRa sensor connection modules. LoRa technology with a long transmission range is used for collecting data from multiple sensors and transmitting the data to the gateway. TVWS technology with high-bandwidth is utilized for transmitting the videos or images from UAVs equipped with special optical sensors. The communication of IoT base stations is introduced in Section 3.3.

The cloud data center is responsible for providing services of data fusion and data analysis. Since the cloud data center is generally far away from the farms, the data from LoRa devices and TVWS will first be forwarded to the gateway. Currently, wired networks or wireless networks are widely deployed in China. According to the “CT China 2008 High Level Forum”, the 4G network covers more than 98% of the population and 95% of the country’s land area of China [48]. Therefore, the gateway can be deployed in farmer houses to forward these data to cloud data centers relying on these networks. In cloud data centers, the data collected by LoRa devices are used for monitoring weather parameters, and the information provided by the TVWS communication system is utilized for generating a precision map and planning the UAV path. It is important to note that the cloud data center is one of the most important parts of the framework and is responsible for processing the data. The results of data analysis will provide the real-time conditions of crops for farmers; details are provided in Section 3.4 and Section 4.2. The system also provides an APP interface, which is more convenient for farmers to control their farms.

### 3.2. Energy Supply Based on an Automatic Rrotary Alignment Device

**Figure 2:**
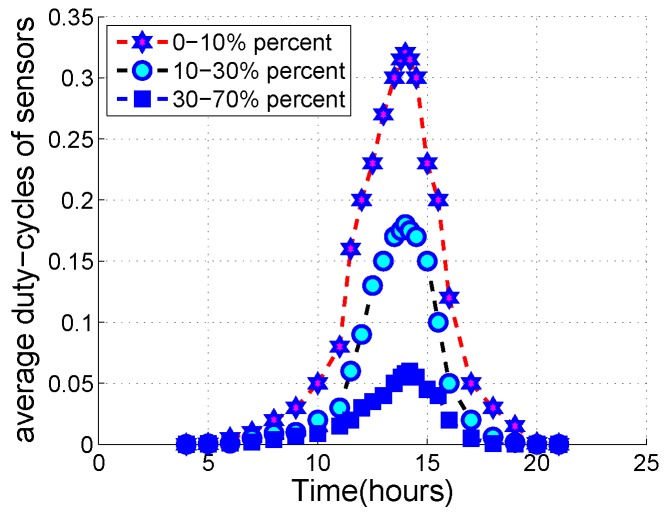
A solar powered sensor is deployed in outdoor.

The energy system uses a solar panel to generate electricity and power sensor devices. The harvesting energy is under the influence of weather conditions. For instance, when a sensor lies in direct sunlight at 12 am, the power density can reach 3700 μW/cm^2^, energy harvesting rate is 370 mW, and the duty cycle achieved by the Crossbow MICAz can reach about 45%. When the sensor node lies indoors, the power density is about 320 μW/cm^2^, the energy harvesting rate is 320 μW, and the duty cycle is estimated to be about 0.04% [49]. Therefore, the energy harvesting sensor networks are environment-dependent networks. Figure 2 shows the affordable duty cycle of a node in three different weather conditions in spring. From the empirical measurement results, a node can only operate about 3.5 h continuously if its duty cycle is over 20%.

**Figure 3:**
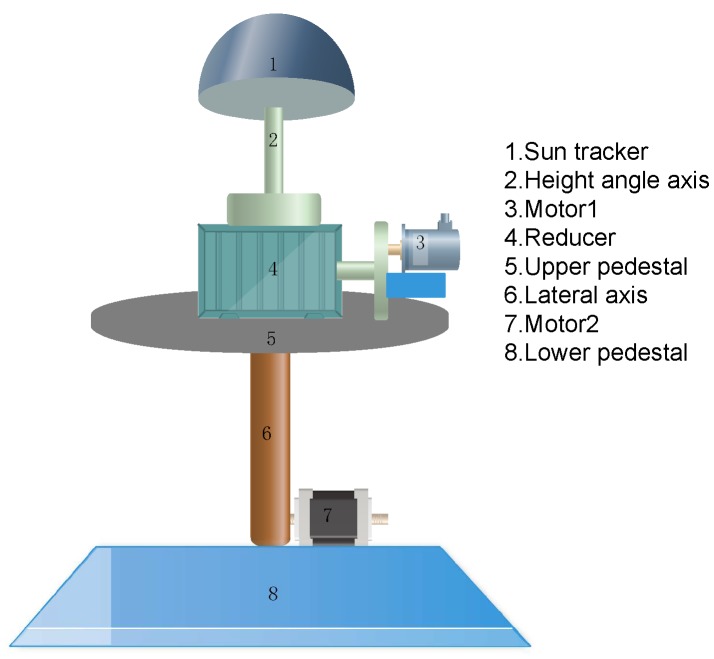
Model of automatic rotary alignment device based on angle perception of sun illumination.

For improving energy-harvesting rates, an OpenWeather API [50] interface is applied in the energy system to obtain the future possible weather in the next few days (e.g., 3 days). According to the obtained information, the system regulates its energy consumption by controlling its duty-cycles. At the same time, an Automatic Device based on Angle Perception of sun illumination, named AD–AP, is used to control a solar panel rotating with the movement of the sun, which guarantees that the solar panel is always perpendicular to the sun. The principle of AD–AP imitates the living habits of sunflowers, which maximize the utilization of solar energy. The AD–AP consists of a sun tracker, a height angle axis, a speed reducer, two motors, an upper pedestal, a lateral axis, and a lower pedestal. The structure of the AD–AP is shown in Figure 3.

To meet the requirement for the solar panels to always be perpendicular to the sun, the turning part of the equipment must satisfy the free rotation of the east–west and north–south directions. At the same time, the principles of low cost, high reliability and simple structure should be achieved. In our design, the automatic solar tracker has double degrees of freedom structure: the translational structure and the lifting structure. Firstly, the tracker analyzes the position of sunlight by obtaining the information from photoresistors. Second, the tracker calculates the angle that it should be adjusted to for harvesting more energy. Finally, the tracker utilizes the translational structure and the lifting structure to change its direction.

**Figure 4:**
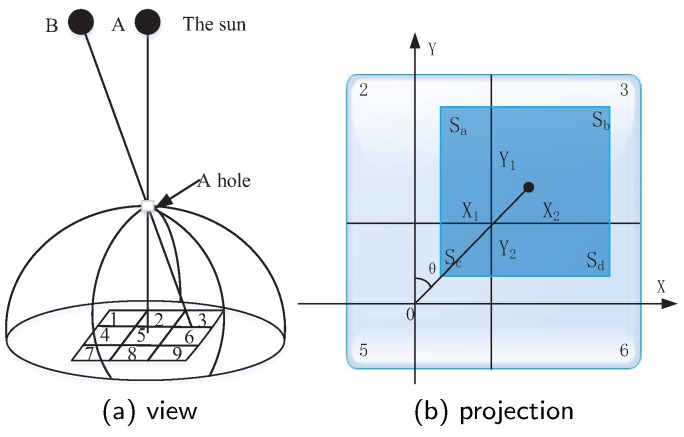
The principle of sun tracking.

The shape of the sun tracker is hemispherical and the entire device adopts an opaque semi-circular outer casing, so that the photoresistor is not affected by the light of the surrounding environment during its operation, which can improve the measurement accuracy significantly. The operating principle of sun tracker is shown in Figure 4a. For allowing sunlight to shine into the panel, a small square hole is left at top of the tracker. Nine photoresistors is placed on the bottom and arrange them in an alignment 3 × 3, where the photoresistor with identifier 5 locates in the center and the size of photoresistor is equal to the size of the hole. The enlarged plan view of the photoresistors is shown in Figure 4b, where, the dark box in the figure indicates the shape in which the sun shines through the top hole on the device. The tracker can detect the sunlight that hits the bottom of the device each few minutes. When the dashed box coincides with the No.5 photoresistor, the device is perpendicular to the sunlight, and vice versa.

Figure 4a plots the scenario of sunlight hitting the photoresistors. When the sun is in position A, the sunlight will shine through the aperture and coincide with resistor 5, which indicates that the tracking device is perpendicular to the sunlight and can harvest more solar energy. While the sun moves to position B, the incoming sunlight obtained by the tracking device deviates from photoresistor 5, which means that the energy harvesting rate declines. For keeping the device perpendicular to the sunlight, the angle of the device needs to be adjusted. We assume that the length of each small square in the figure is *M*, then the area of each small square is $S=M^{2}$.

For calculating the angle that should be adjusted of the sun tracker, in Figure 4b, we assume that the length of four sides for the dark square are $X_{1}$, $X_{2}$, $Y_{1}$, $Y_{2}$, respectively. The four areas of the dark square are $S_{a}=X_{1}\times Y_{1}$, $S_{b}=X_{2}\times Y_{1}$, $S_{c}=X_{1}\times Y_{2}$, $S_{d}=X_{2}\times Y_{2}$. The value of each small area in the system can be obtained based on the amount of information returned by all photoresistors after sunlight exposure. The amount of information for each small area is expressed as: $X_{a}$, $X_{b}$, $X_{c}$, $X_{d}$. The following relationship can be obtained: $X_{a}=S_{a}/S$, $X_{b}=S_{b}/S$, $X_{c}=S_{c}/S$, $X_{d}=S_{d}/S$. Moreover, it is known that $X_{1}+X_{2}=Y_{1}+Y_{2}=M$. Therefore, $X_{2}$, $Y_{1}$ can be expressed as:

(1) $$\begin{array}{c} X_{2}=\frac{(S_{b}+S_{d})\times M}{S} \end{array}$$

(2) $$\begin{array}{c} Y_{1}=\frac{(S_{a}+S_{b})\times M}{S} \end{array}$$

Depending on Equations (1) and (2), the θ can be calculated as,

(3) $$\begin{array}{c} \theta =\arctan\frac{X_{2}}{Y_{1}} \end{array}$$

**Figure 5:**
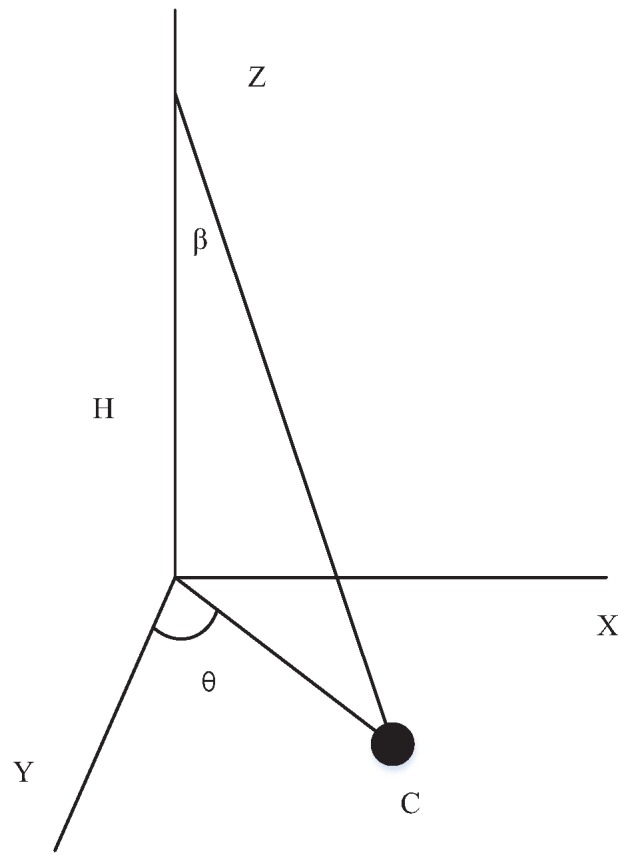
A three-dimensional coordinate when the device is not perpendicular to the sunlight.

For adjusting the direction of the sun tracker, the graph is switched into three-dimensional coordinates in the sphere for their benefit, as shown in Figure 5. The dot in Figure 4b is point C in Figure 5, and *h* is the distance from the bottom of the device to the apex of the device. We have

(4) $$\begin{array}{c} \tan\beta =\frac{\sqrt{X_{2}^{2}+Y_{1}^{2}}}{h} \end{array}$$

where, β can be formulated as:

(5) $$\begin{array}{c} \beta =\arctan\frac{\sqrt{{\left(\frac{(S_{b}+S_{d})\times M}{S}\right)}^{2}+{\left(\frac{(S_{a}+S_{b})\times M}{S}\right)}^{2}}}{h} \end{array}$$

For keeping the AD–AP perpendicular to the sunlight, the device can first move *θ* angle in the X-axis, and then move *β* angle in the opposite direction of the Z-axis to ensure that the device is perpendicular to the sunlight again. In order to save energy, the direction of the tracker should be adjusted back to the initial state at night and then remain unchanged. On cloudy days, the tracker does not need to rotate for saving energy. After the tracker adjusts its position, a reasonable period of time is set to initiate another adjustment, e.g., ten minutes or one hour, etc. In some special situations, if the tracker can not adjust to a normal position in the period of time, it can be considered that the remaining energy of AD–AP can not support its action, e.g., the weather is cloudy or rainy. In this situation, the tracker should try to adjust the position after another period of time until the sun tracker is perpendicular to the sunlight again.

### 3.3. Communication Systems of the Agricultural IoT Platform

In the design, the communication system contains two parts: LoRa technology and TVWS technology. LoRa technology is a point-to-point communication mode with low power consumption, low data transmission rate and long transmission range, where, the transmission distance can reach 15 km in the unobstructed situation, while the transmission range is only about 100 meters for ZigBee devices (e.g., MICAz, TelosB) [51]. As a gateway, a LoRa device can connect a certain number of wireless sensor nodes for data collection, which replaces the traditional General Packet Radio Service (GPRS) module (monthly fee, e.g., about 2 dollars every month in China). Therefore, in the long run, LoRa technology can effectively reduce communication costs by replacing GPRS as the gateway.

**Figure 6:**
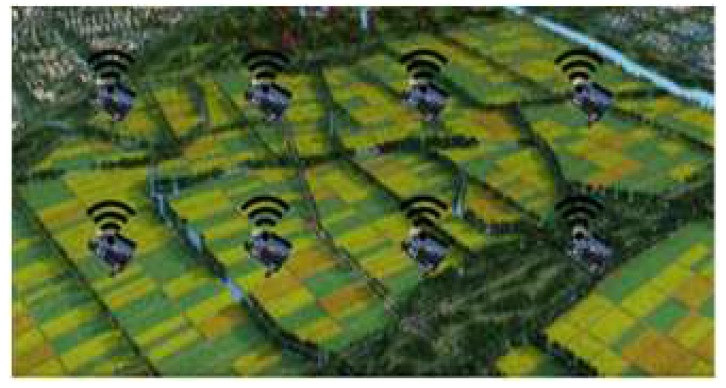
Multiple LoRa devices are deployed in farmland.

When LoRa devices are used as relay nodes in a multi-hop LoRa mode, data can reach hundreds of kilometers through multiple-hop forwarding at low rates without the help of satellites or base stations, which is sufficient for data transmission in modern agricultural fields. In some countries, LoRa technology has been employed in some agricultural cooperations, e.g., intelligent irrigation systems [52]. LoRa technology is utilized for collecting information by connecting the sensors deployed in the farm, and transmitting the collected data to the base station/gateway. In the system, multiple LoRa devices are deployed in the farm, as is shown in Figure 6. A base station or gateway generally is equipped with high energy power and transmission ability. Thence, all packets will be forwarded to farmer’s home by multiple gateways. For processing received data, the cloud data center is built and stores all collection data. However, the cloud data center is usually far away from the farmer’s home, and there are many obstacles between them to hinder the transmission of information. It is dificult to accomplish the task of data transmission relying using traditional methods (e.g., ZigBee, LoRa, Bluetooth).

The White Space is a blank TV signal band, which refers to a radio video segment that is allocated for broadcast utilization but is out of use. It has the advantages of large user volume and low cost. TVWS has also been applied internationally. For example, the United States is actively deploying farm super Wi-Fi by virtue of TVWS technology [53]. Due to its low frequency and support for high-bandwidth data transmission, TVWS technology fully meets the requirement of high-bandwidth connections between UAVs and gateways. In China, due to the popularity of digital TV, traditional wireless TV has gradually faded out of sight, which makes the TVWS band free, and can fully utilize this band for high-bandwidth data transmission.

**Figure 7:**
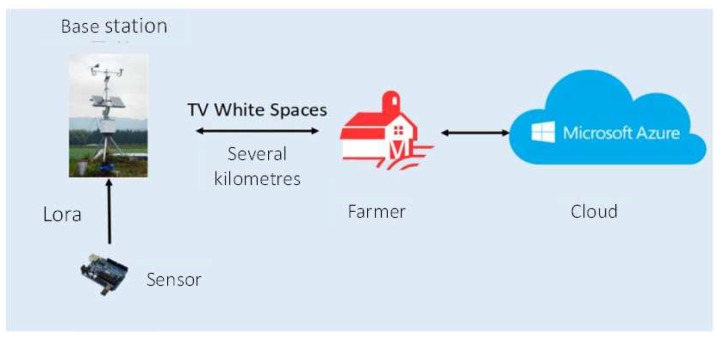
A two-layers data communication architecture from sensors to farmer.

In the communication system, on the one hand, the compatibility of distinct communication technologies (e.g., ZigBee, LoRa, TVWS) should be guaranteed. On the other hand, the requirement of long-distance communication and the high-bandwidth connection should be achieved simultaneously. Therefore, a hybrid network based on two-layers for data transmission is adopted in the work. At the first layer, TVWS is used to connect the system of farmers’ homes with IoT base stations on the farm. In the second layer, the LoRa module with long transmission distance and strong anti-interference ability is used to achieve information collection between the base station and sensors deployed in farmland, which ensures that a minimum of sensor nodes can check the maximum farm area. Data communication architecture is shown in Figure 7. Currently, a wired network or wireless network is widely deployed in farmers’ houses of China. Hence, the existing Wi-Fi infrastructure can be exploited, and these existing Wi-Fi devices can be employed to undertake the tasks for data forwarding from the farmer to the cloud data center.

### 3.4. The Path Planning of Unmanned Aerial Vehicle

In the design, the UAVs are used to detect the pests and diseases on the farms by periodically capturing the images of crops with a spectral camera. Afterwards, these images will be transmitted to the cloud data center and stored for data analysis. With the aid of image processing technology, these images can be analyzed to determine whether there are pests and diseases or not. Currently, a UAV usually is equipped with a smaller fuel tank, which causes the flight time to be short, so that the flight time of a UAV is generally about 30 min in China. For improving energy utilization efficiency, the flight path of the drone is planned previously. When UAVs take photographs on farms, they fly from the initial point to the target point automatically depending on the planned paths. Therefore, it is crucial to plan the flying routes for prolonging the flight time. At the same time, the planning paths are restricted by height, the field of view of the camera, and the required image quality, etc.

Multiple flight paths can be designed to check the entire farmland, where one of them is preferred and other paths are set as potential flying paths. Intuitively, for a UAV, the shortest path is the best choice to achieve the longest flight time. However, considering weather conditions, especially factors such as wind speed and wind direction, the planning path with the highest proportion of downwind can ensure the maximum utilization of wind force. At the same time, during the flight, the speed of the drone can be adjusted dynamically to save energy and extend the flight time of a UAV. When a drone flies on the farm, without considering the wind force, if the flight speed of the drone is $V_{w}$ and the flight distance is *S*, the flight time can be calculated as:

(6) $$\begin{array}{c} t=\frac{S}{v_{w}} \end{array}$$

**Figure 8:**
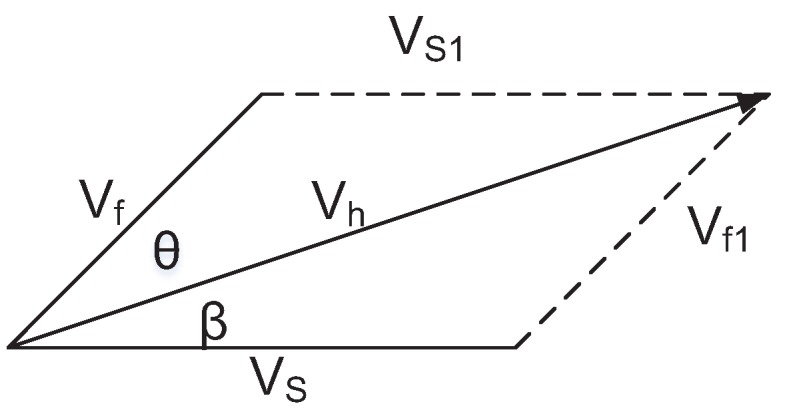
When consider the wind speed and wind direction, how to control the flight speed and direction of UAV.

The farmlands usually cover large areas and are open, where wind force exists commonly. When a drone flies in such a scenario and the wind force has not been utilized efficiently, it may cause unnecessary energy waste and reduce the flight time. Therefore, considering the wind force on the farm, a flight mode is designed to save energy and prolong the fight time of UAVs. When there is an angle between the wind direction on the farm and the planning path of the drone, as shown in Figure 8, if the flight time of the drone is required to be held in this planning path, or in other words, if the original energy expenditure rate is required to remain constant, the flight speed and flight direction of the drone should be adjusted dynamically.

The drone is equipped with special sensors for acquiring the wind direction and wind speed. Thus, the $V_{f}$ is available depending on the surface sensor in the drone. The symbols that used in Figure 8 are summarized as in Table 1.

According to Equation (6), the equality $V_{h}=V_{w}$ is achieved. For calculating the $V_{s}$ and β, we find that $V_{s}=V_{s1}$, $V_{f}=V_{f1}$. Therefore, $V_{s1}$ can be formulated as:

(7) $$\begin{array}{c} V_{s1}=\sqrt{V_{f}^{2}+V_{h}^{2}-2\times V_{f}\times V_{h}\times \cos\theta } \end{array}$$

According to Equation (7), the *β* angle is expressed as:

(8) $$\begin{array}{c} \beta =\arccos\frac{V_{h}^{2}+V_{s}^{2}-V_{f}^{2}}{2\times V_{f}\times V_{h}} \end{array}$$

According to Equations (7) and (8), the direction and the speed of the drone in actual flight are known. If the energy consumption is expected to be reduced by the drone, the speed of the drone should satisfy $V_{s}<V_{w}$. Since $V_{w}=V_{h}$, we can find that inequality $V_{s}<V_{h}$ holds. According to Equations (7) and (8), in order to save energy of a drone, the following constraints need to be satisfied:

(9) $$\begin{array}{c} V_{f}<2\times V_{h}\times \cos\theta \end{array}$$

Although the wind speed and wind direction on a farm can not be controlled artificially, the angle between the wind and the flight path can be controlled. At the same time, the flight speed of drone can be adjusted dynamically according to the wind speed. Therefore, it is necessary to design the flight path of the drone in combination with the actual conditions. It is interesting to note that it is a challenge for the stability of a drone in some special scenarios with strong wind. However, note that we would only require long flight times when the pests and diseases occur in farms and we only use the UAV to collect the information of crops. Hence, a sunny day can be selected to arrange the flight or in a day with slight wind, which is different than if the UAV is used in military fields with time-tight requirements.

## 4. Analysis

### 4.1. Energy Harvesting Analysis

**Figure 9:**
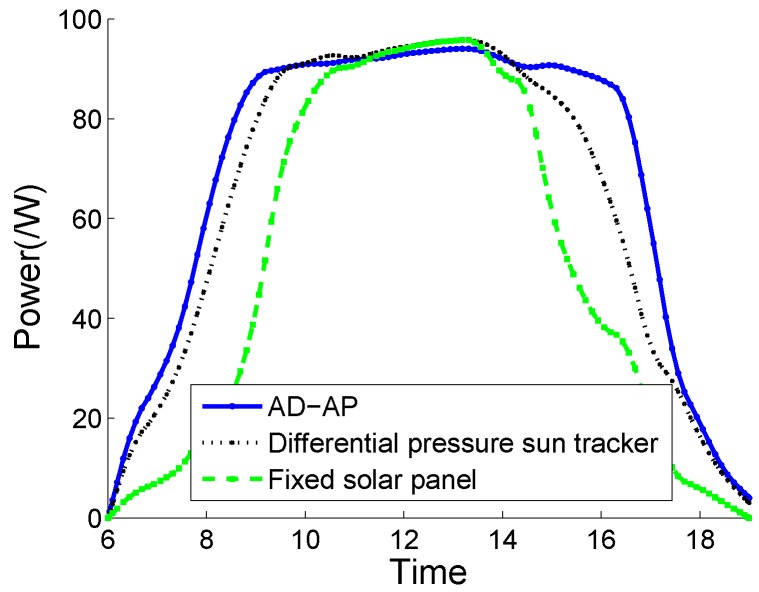
Comparison of energy harvesting power of three devices.

A solar panel, two eZ430-RF2500T target boards and one AAA battery pack is used for outdoors, which is rechargeable and can be recharged repeatedly. The target board comprises the TIMSP430 microcontroller, CC2500 radio transceiver and an on-board antenna. The CC2500 radio transceiver operates in the 2.4 GHz band with data rate of 250 kbps and is designed for low power wireless applications. The harvested energy is stored in EnerChip, a thin-film rechargeable energy storage device with low self-discharge manufactured by Cymbet. Figure 9 plots the energy harvesting power of our design, a differential pressure sun tracker device [54], and a fixed solar panel. For the fixed solar panel, it means that the solar panel is fixed and it does not whirligig with time. The superiority of the fixed solar panel model is that the energy for powering the motor to whirligig the solar panel is saved.

Based on the fixed solar panel, the optimal energy acquisition power in the sunrise and sunset time range of the Yangtze River basin in mid-March was tested. From Figure 9, the harvesting energy based on a fixed solar panel is higher than the other two schemes from 11 am to 1:30 pm. The reason is that extra energy is not needed to power the fixed solar panel. While before 11 am and after 1:30 pm, for the fixed solar panel, the harvesting energy is lower than AD–AP and the model of [54]. According to the investigation, the differential pressure solar tracker [54] can use the pressure difference of the medium in the container to slowly adjust the device back to a state perpendicular to the sunlight. This tracker has a simple structure, but it is limited to a single-axis tracker with low precision. From Figure 9, the incident angle between the sunlight and the fixed solar panel is close to 0 and the energy acquisition power is at a maximum from 11:00 to 14:30. The installation of an automatic rotary alignment device proposed in our system enables the energy harvesting power to reach its peak value at around 9 o’clock in advance, and can increase the energy acquisition power by about 38% in one day.

### 4.2. Generating Accurate Maps for Farms

For detecting the emergence of pests and diseases in agriculture precisely, it is necessary to analyze the data of the accurate farm maps taken by a drone, which means that the entire farm has a specific distribution of characteristics based on image analysis. The agricultural IoT platform supports a novel and accurate map generation method that utilizes the aerial imagery of drones and the data returned by sensors placed on the farm. Specifically, the system uses the images generated by the drone video and the values observed by the sensors planted in the soil to predict the possibility of occurrence of diseases and pests. The system’s gateway embeds a machine learning pipeline that draws on probabilistic graphical models that embed Gaussian processes [55].

**Figure 10:**
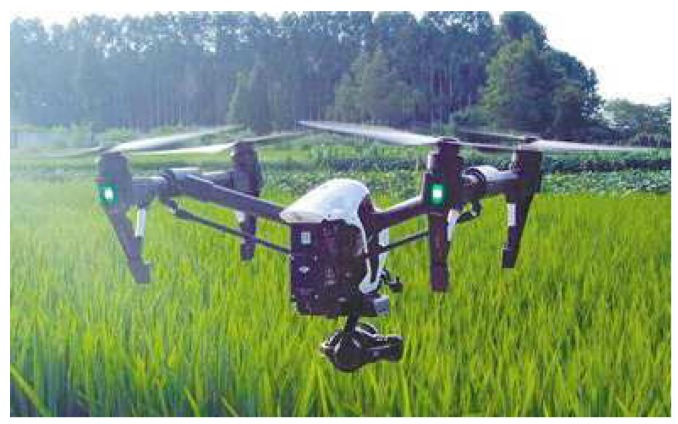
Hyperspectral sensor on-board a DJI T600 unmanned aerial vehicle (UAV).

Hyperspectral imagery was acquired using a HySpex ODIN-1024 (Norway), as shown in Figure 10. The hyperspectral sensor recorded data cubes of 427 spectral bands in the visible and near-infrared (VNIR) range (400–2500 nm) with a 3 nm spectral interval and a 6.1 nm spectral resolution (full width at half maximum (FWHM) with 10 μm slit). This camera is equipped with a calibrated f/1.8 4.8 mm Schneider lens, which results in a 50.7 deg field of view over 1024 pixels. The collected hyperspectral data cubes are synchronised with a GPS/inertial navigation system (INS) positioning and orientation information in order to perform data cubes orthorectification and multiple data cube mapping; the detail of HySpex ODIN-1024 can be seen in [56].

**Figure 11:**
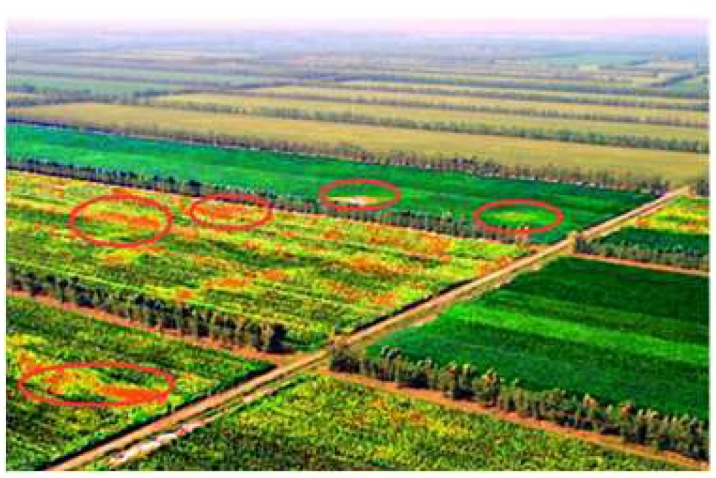
The analysis results of pests for a UAV image.

The key to this model is visual and spatial smoothness. For visual smoothness, since the monitoring area presents spatial continuity, it indicates that the monitoring areas that look similar have identical values of weather paraments and possibility of pests, such as a recently irrigated area looking darker and it can be easily inferred that this area has more moisture. For spatial smoothness, considering the physical properties of the soil and the environment, the sensor readings in the nearby area will be similar. The map generated by the above technique is shown in Figure 11.

In our design, the system uses accurate maps as units of data aggregation and sends them to the cloud data center. There are at least two advantages to using this approach. Firstly, it integrates sensor data from the farm into drone videos. Secondly, it can be compressed to two or three orders of magnitude, much smaller than directly transmitting images. Consequently, aerial imagery is suitable for providing farmers with a detailed overview of the farm, while accurate maps are more suitable for long-term storage and transmission.

### 4.3. Pests and dIseases of Crops Are Analyzed Through Reflection Spectrum

**Figure 12:**
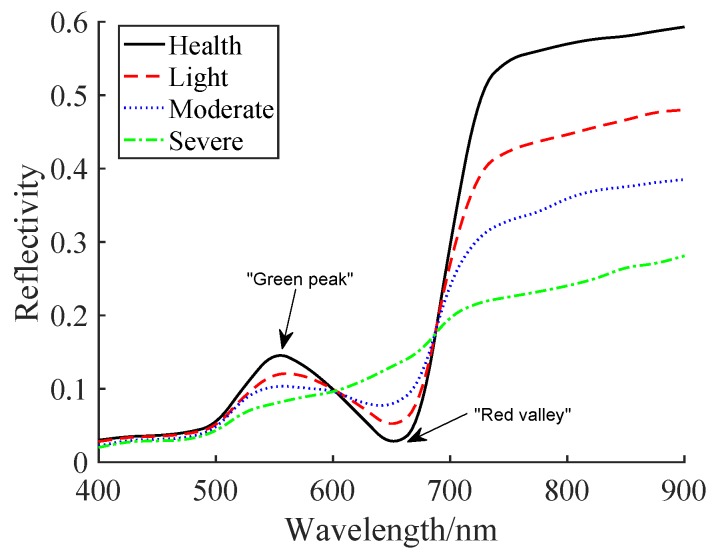
Spectral characteristics of wheat under different degrees of disease.

If crops are infected by pests and diseases, their coverage, biomass, Leaf Area Index (LAI), leaf cell structure, nitrogen, moisture, pigment content, and appearance will change, which leads to changes in the reflectance spectrum of the visible to thermal infrared spectrum. In particular, the spectral characteristics of the infrared and red regions are different from those of healthy crops. Then, by monitoring the reflectance spectrum of crops, whose disease statuses can be obtained. Figure 12 shows the spectral characteristics of healthy and diseased wheat. The spectral reflectance of healthy wheat produces a trough in the red region (“red valley”) due to the large amount of radiation absorbed by chlorophyll. In the green zone, the absorption of chlorophyll is reduced, resulting in a robust green reflection zone (“green peak”).

Regarding quantitative analysis, the relationship between chlorophyll content and spectral response, red edge parameters area of red edge and position of red edge in the first derivative of reflectance curve were obtained at bands of 680–760 nm. Similarly, green peak and red valley parameters were defined to reflect spectral character. The detail of red valley and green peak is introduced in [57]. As can be seen from Figure 12, as the degree of disease continues to increase, the spectrum changes significantly. It can be clearly found that the “red valley” in the red light range and the “green peak” in the green light range gradually disappear. In the near-infrared region, the spectral reflectance of infected wheat is significantly lower than that of healthy wheat. Through the analysis of the spectrum, the degree of damage of pests and diseases can be monitored to provide timely and accurate information for pest control.

**Figure 13:**
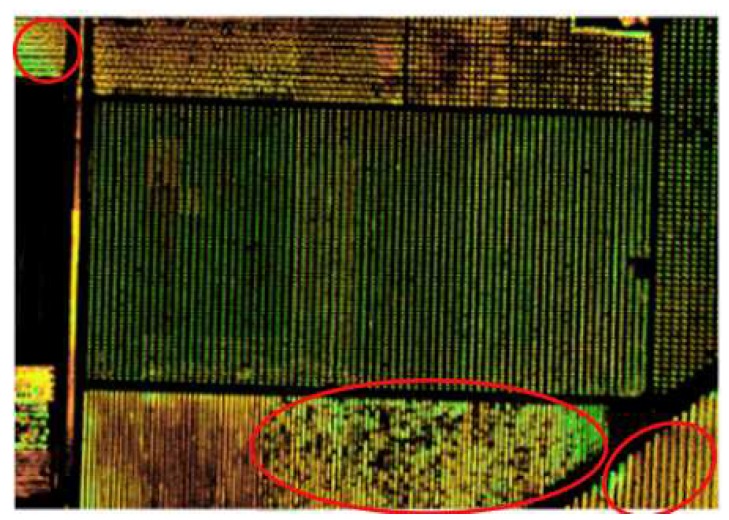
Low-altitude remote sensing image of pest monitoring.

Depending on the image processing technology, aerial pictures can be used to detect the occurrence of pests and diseases in crops. As shown in Figure 13, the crop is suffering from the disease, and the yellower the picture, the more serious the disease; the greener, the lighter the disease. Through sensors deployed in the farmland, the comprehensive environment around the crops can be analyzed, the temperature is 17 °C, the relative humidity is 68%, the wind speed is 2.3 m/s, and the light intensity is 554 W/m^2^. After experiments and analysis of the above factors, if the crops grow in this environment or a relatively closed environment, the crops have a higher probability of occurrence of pests and diseases.

### 4.4. Relationship between Pests/Diseases and Weather Parameters

The real-time monitoring technologies of pests and diseases are generally divided into two categories: indirect monitoring schemes and direct monitoring schemes [58]. A direct monitoring scheme is by analyzing the images of crops through the reflection spectrum of plants [59], while an indirect monitoring scheme is to analyze the weather parameters and get the probability of crop pests and diseases [60]. Generally, in a special temperature range, the growth rate of most pests is accelerated and the growth cycle of pests is shortened with temperature increasing, and vice versa [61]. Temperature also affects the number of pests, migration, reproduction, and longevity. Rain and humidity are other key factors leading to the occurrence of pests and diseases. Studies have shown that the increasing rainfall in spring and winter leads to the prevalence of powdery mildew of wheat in the Yangtze River region of China [48].

As another critical factor, wind force also affects the emergence and diffusion of pests and diseases. Relevant surveys have shown that the prevalence of wheat powdery mildew in the Yangtze River basin is positively correlated with the average wind speed during wheat growth. Through the comprehensive analysis of the surrounding environment of the above crops, the probability of pests and diseases of crops can be obtained. From April to May of 2017 in China, the temperature in most winter wheat regions was close or slightly higher than that of 2016. In the northern Huang-Huai-Hai, southern North China and most southwestern wheat regions, the temperatures were 0.5 °C–1 °C higher than those of 2016, which provided favorable environmental conditions for the occurrence and reproduction of aphids and pathogens such as powdery mildew and sheath blight in the region.

**Figure 14:**
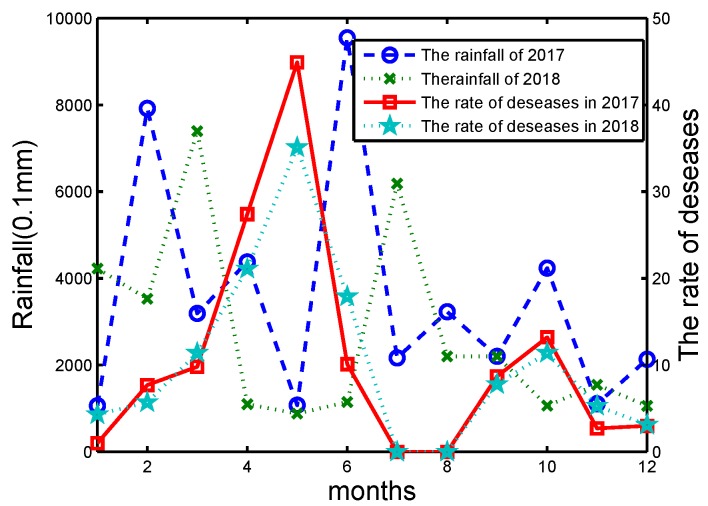
The rainfall and wheat incidence in Yangtze River Zone of China from 2017 to 2018.

**Figure 15:**
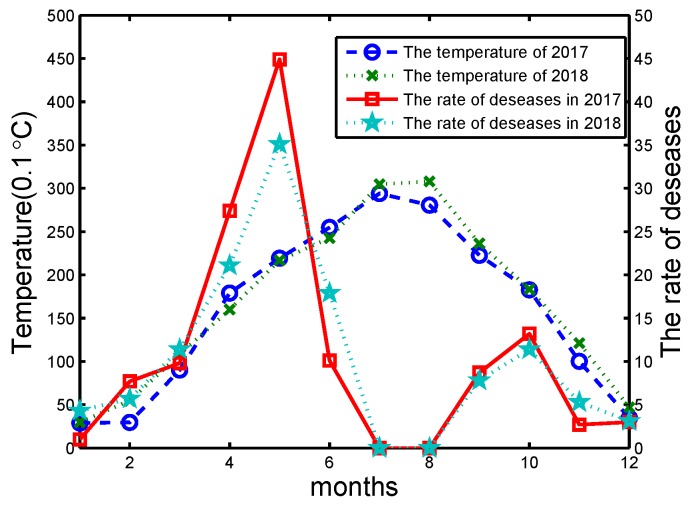
The temperature and wheat incidence in Yangtze River Zone of China from 2017 to 2018.

In 2017, the total area of wheat aphids in China was about 250 million acres, which mainly contains the Huang-Huai-Hai agricultural region and the Yangtze River region. Powdery mildew takes over about 120 million acres, and sheath blight has a cumulative area of about 90 million acres. Depending on the data obtained by the sensors in the farm, the relationship between the occurrence of pests/diseases and the farm environment is analyzed deeply. Take wheat powdery mildew as an example in Yangtze River Zone of China from 2017 to 2018. The Yangtze River is the largest river and the Yangtze River Zone has a large population and is one of the most economically developed areas in China, which is also a major food producing area. The main environmental factors affecting wheat powdery mildew were analyzed during the critical period of wheat growth from sowing to maturity. The results are shown in Figure 14 and Figure 15.

During early spring (April to May), the temperature ranges from 15 °C to 20 °C and is favorable for growing crops. At the same time, the temperature is beneficial to the occurrence of pests and diseases. From Figure 15, the risk of pests increased with temperature and rainfall increasing significantly, and vice versa. In summary, when the temperature is low and precipitation is high, the weather condition is not conducive to wheat growth. The wheat will grow slower and is easily invaded by pests and diseases.

**Figure 16:**
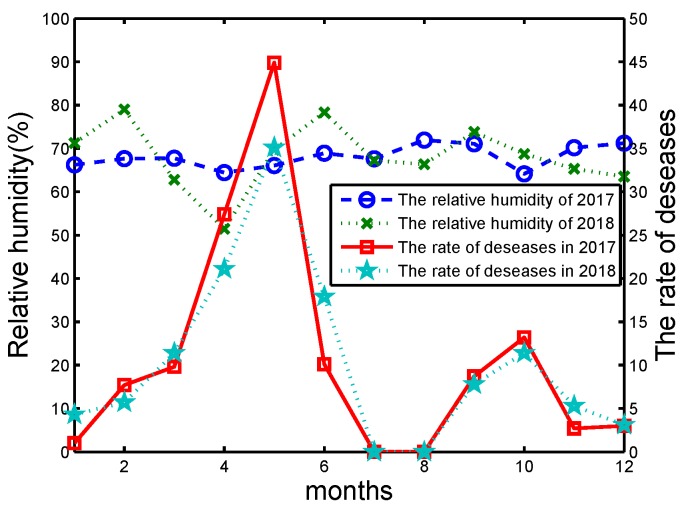
The relative humidity and wheat incidence in Yangtze River Zone of China from 2017 to 2018.

If the humidity is considered for the occurrence of diseases, the spores of pathogens can germinate in the humidity ranging from 0% to 100%. Higher humidity is more favorable for the spread of bacteria. When the relative humidity reaches 65% or more, it may cause a massive outbreak of wheat powdery mildew, as shown in Figure 16. If the rainfall is large and concentrated, it can destroy the pathogens that are parasitic on wheat.

**Figure 17:**
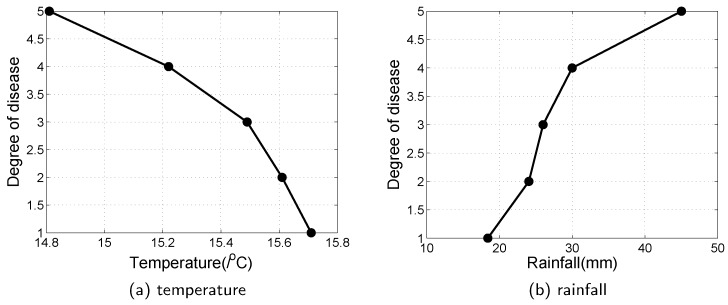
Effect of spring temperature and rainfall on the occurrence of disease.

For measuring the disease damaging degree to crops, a degree of growth condition of the crops is applied in this work, where level 0 indicates crop health, level 1 indicates that the symptoms of the disease are not obvious, and level 2 indicates obvious symptoms on leaves or straw, but the area of the disease does not exceed 50%. Level 3 indicates apparent symptoms on the leaves or straw, and the area of the disease is more than 50%. Level 4 indicates that the whole plant is damaged and level 5 indicates that the rot occurs. We note that this measurement is widely used in agriculture fields for judging the growth condition of crops and the detail of it is shown in [62]. Precipitation and temperature are related to the occurrence and prevalence of wheat powdery mildew. Wheat is susceptible to this disease when the temperature is between 14 °C and 16 °C. In this temperature range, the germination rate of conidia is the highest and the mycelial growth rate is also the fastest. Temperature affects the rate of propagation of fungal spores, and rainfall affects the spread of wheat powdery mildew, as shown in Figure 17.

## 5. Conclusions And Future Planning

In this work, for providing energy to our IoT framework in remote farms, an automatic rotary-device based on angle perception of sun illumination is added for improving the utilization of solar energy, prolonging the use time of the sensor, and ensuring that the data collected by the sensor are valid in real time. The main contributions for the aspect of drones in this article are: (i) planing the flight route of the drone, (ii) adjusting the flight speed of the drone, (iii) making full use of the farm wind force, and (iv) extending the flight time of the drone to meet the low-altitude remote sensing demand for pests and diseases on large outdoor farms. It represents the macro and micro perspectives of modern agricultural techniques of low-altitude remote-sensing technology and IoT technology for monitoring pests and diseases of crops.

Through the analysis of large amounts of data obtained by drones and sensors, we provide an insight into the specific relationship between the occurrence of pests and the farm environment. The prevention and control of agricultural pests and diseases is a systematic project and a significant challenge for farmers and researchers; it requires long-term observation and analysis using information technology. Through more extended data accumulation and analysis of large amounts of data, a long-term pest and disease prediction model will be established. Based on agriculture, the model proposed in this work will be verified in practical applications. Note that, on the one hand, the pests and diseases of crops can be monitored in real time based on the framework. On the other hand, the occurrence of pests and diseases can be studies by analyzing climate changes, and some precautions against pests can be implemented in advance. 

## Figures and Tables

**Table 1:** Symbol List of Figure 8.

Symbol	Description
$V_{f}$	the wind speed on the farm
$V_{h}$	the actual flight speed, which combination of
	the speed of drone and the wind
$V_{s}$	the planing flight speed of the drone
θ	the angle between $V_{f}$ and $V_{h}$
β	the angle between $V_{s}$ and $V_{h}$
